# Validating the Children’s Sleep Habits Questionnaire Against Polysomnography and Actigraphy in School-Aged Children

**DOI:** 10.3389/fpsyt.2014.00188

**Published:** 2015-01-06

**Authors:** Adria Nora Markovich, Melissa Anne Gendron, Penny Violet Corkum

**Affiliations:** ^1^Department of Psychology and Neuroscience, Dalhousie University, Halifax, NS, Canada; ^2^IWK Health Centre, Halifax, NS, Canada; ^3^Colchester East Hants ADHD Clinic, Truro, NS, Canada

**Keywords:** validation, CSHQ, sleep, polysomnography, actigraphy

## Abstract

Sleep is a vital physiological behavior in children’s development, and as such it is important to be able to efficiently and accurately assess whether children display difficulties with sleep quality and quantity. The Children’s Sleep Habits Questionnaire [CSHQ; (1)] is one of the most commonly used assessment tools for pediatric sleep. However, this instrument has never been validated against the gold standard of sleep measurement [i.e., polysomnography (PSG)], and studies comparing it to actigraphy are limited. Therefore, the current study assessed the validity of four subscales of the CSHQ via direct comparison with PSG and actigraphy for 30 typically developing school-aged children (ages 6–12). No significant correlations between relevant CSHQ subscales and PSG variables were found. In terms of the actigraphy variables, only the CSHQ Night Wakings subscale achieved significance. In addition, sensitivity and specificity analyses revealed consistently low sensitivity and high specificity. Overall, the CSHQ Sleep Onset Delay, Sleep Duration, Night Wakings, and Sleep Disordered Breathing subscales showed low construct validity and diagnostic validity. These results underscore that caution should be taken when using the CSHQ as the sole screening tool for sleep problems in children.

## Introduction

Sleep is essential to children’s physical and emotional well-being. Unfortunately, recent studies show an increasing trend toward decreased sleep in childhood due in part to progressively later bedtimes and unchanged wake times (2, 3). School-aged children have greater sleep needs than adolescents and adults, and typically require 10–11 h of sleep/night (4). Today, the average child is not meeting this recommended duration (5, 6), which can have a range of daytime consequences.

Sleep difficulties, both in terms of the quantity and quality of sleep, can have a significant impact on children’s daytime functioning. Vriend et al. (7) found that poor sleep in typically developing children was associated with consequences such as increased negative affect (i.e., higher levels of sadness and anger) and impaired attention. These same researchers also assessed the effect of controlled manipulations of children’s sleep on daytime functioning by shortening and lengthening children’s typical sleep duration by 1 h for 4 days (8). Sleep restriction resulted in significant difficulties with emotion regulation, less positive affect, weaker attention, and lower academic productivity. These findings indicate that even a small cumulative change in sleep can have significant daytime consequences.

It is clear from previous research that many facets of children’s daytime functioning, including emotional health, interpersonal relationships, and academic performance, are negatively affected by poor sleep (7–9). These implications are sobering when one takes into account the vast number of children presently suffering from sleep difficulties. Sleep problems affect approximately 25% of all children, with some prevalence estimates reaching upwards of 40% (10). As such, accurate measurement of sleep is important, as this allows for assessment of whether or not a child’s sleep needs are being met.

There are various ways to measure sleep in children, through the use of methods that range in their degree of objectivity and subjectivity. Polysomnography (PSG) is considered to be the gold standard sleep measurement (4), against which other measures are typically tested [e.g., (11–16)]. Nocturnal PSG involves directly monitoring a child’s overnight sleep, and typically occurs in a sleep lab or hospital setting. Electrodes are placed at specific locations on a child’s scalp, face, neck, chest, and legs, which provide continuous electrophysiological recordings of brain activation, eye movements, skeletal muscle activation, and heart rate. In addition, piezo chest bands, nasal cannulas, and pulse oximeters assess respiratory effort, airflow, and oxygen saturation. These electrophysiological data provide a range of information about sleep, including how long it took a child to fall asleep, the total time spent asleep, and how well the child slept overall. In addition, the data provide information about nocturnal awakenings, limb movements, sleep architecture (e.g., the amount of time spent in REM and non-REM sleep), and any sleep-related breathing difficulties (17).

Polysomnography provides very robust, objective data; however, because this method of assessment requires placement of electrodes by a specialist, and usually requires that a child stay overnight in a sleep lab, there is a degree of inconvenience and cost associated with this measure (18). In light of this, actigraphy is becoming an increasingly popular method of assessing sleep in children. Allowing for sleep measurement within a child’s home setting, actigraphy is less costly and less invasive than PSG. (However, it should be noted that the expense is not negligible, as a single actigraph currently costs upwards of $1,000). An actigraph (commonly in the form of a wristwatch-like device) uses an accelerometer to record gross motor activity in order to estimate sleep–wake cycles. A child remains in their home environment and wears an actigraph on their non-dominant wrist, usually for a period of days or weeks. Activity data are collected and stored by the actigraph until the testing period is complete, at which point the information is downloaded and analyzed (17). Actigraphy data provide information about the length of a child’s sleep, whether they experienced any awakenings, and how efficient their sleep was overall.

Unlike PSG, actigraphy has a subjective component associated with it, as sleep diaries are needed in order to determine several sleep variables. Sleep diaries are daily sleep logs completed by parents (and/or the child), in which various aspects of a child’s sleep behaviors are recorded. From these sleep diaries, parent- and/or child-indicated bedtimes and wake times are required to score actigraphy variables such as the total time spent in bed, and how long it took the child to fall asleep. Such scoring of actigraphy data also involves a degree of clinical judgment on the part of researchers and clinicians, which may lend an additional level of subjectivity to this measure. Moreover, actigraphy only estimates sleep based on body movement and does not record brain activity. As such, actigraphy cannot determine sleep architecture. Other variables recorded by PSG are also not recorded by actigraphy, such as respiration, oxygen saturation, and heart rate. While actigraphy allows for less invasive measurement of children’s sleep, it only provides an estimate of a limited range of the variables collected via PSG.

Although objective measures of children’s sleep behavior produce highly reliable and valid data, the cost, time, and effort associated with these measures can also make them difficult to administer on a wide scale (18). Clinicians frequently opt to use questionnaires instead of PSG and/or actigraphy because of their time- and cost-effective nature, as well as their relative ease of administration. While sleep diaries are also a less costly measure, they are time-consuming to complete, and the results are difficult to interpret, as there are no norms that exist for comparison. Questionnaires involve subjective (and typically retrospective) ratings of a child’s sleep behaviors. A child or parent rates the frequency of various sleep behaviors, or indicates their agreement with a number of statements pertaining to the child’s sleep. In completing the questionnaire, the child or parent is typically instructed to focus on a specific period of time (e.g., the previous week or month). In addition to their ease of administration and scoring, sleep questionnaires have norms for comparative purposes, and as such are widely used. This widespread use has led many researchers to assess the validity of sleep questionnaires via comparisons with objective measures of sleep behavior such as PSG (11, 19, 20), actigraphy (21–23), or both (12, 13). In general, these results have been inconsistent.

One of the most commonly used sleep screening questionnaires for school-aged children is the Children’s Sleep Habits Questionnaire (CSHQ), developed by Owens et al. (1). The CSHQ’s ubiquity is evidenced by its widespread use in both the research and the clinical community. At the time of the current study, the CSHQ has been cited in over 600 published journal articles. Moreover, in a clinical capacity, it is one of the most common tools used for assessing sleep problems in children. The CSHQ is a 45-item, parent-rated questionnaire that assesses the frequency of behaviors associated with common pediatric sleep difficulties. A retrospective measure, the CSHQ instructs parents to rate the frequency with which their child has displayed various sleep behaviors during the previous week. Ratings are combined to create eight subscales that relate to common sleep problems in children: Bedtime Resistance, Sleep Onset Delay, Sleep Duration, Sleep Anxiety, Night Wakings, Parasomnias, Sleep Disordered Breathing, and Daytime Sleepiness. Finally, all ratings are summed to create a Total Sleep Disturbances index, for which a score of over 41 has been found to indicate a pediatric sleep disorder (1).

During the process of developing this scale, Owens et al. (1) assessed CSHQ ratings for a community sample of 469 school-aged children and a clinical sample of 154 children previously diagnosed with pediatric sleep disorders. The clinical sample was made up of three groups of children who had received a diagnosis of either a behavioral sleep disorder, parasomnia (e.g., sleep walking, night terrors, etc.), or sleep-disordered breathing in a pediatric sleep clinic. The CSHQ was found to have acceptable internal consistency of 0.68 and 0.78 for the community and clinical samples, respectively. A second CSHQ was completed by 60 parents in the community sample, and the test–retest reliability of the subscales ranged from 0.62 to 0.79, which was considered to be acceptable. Finally, the validity of the CSHQ was investigated by comparing total scores and subscale scores of the community and clinical samples. The three groups in the clinical sample had significantly higher total scores and subscale scores than the community sample, and this ability of the CSHQ to differentiate between children with and without sleep disorders was taken as evidence of the questionnaire’s validity. The sensitivity and specificity of the CSHQ were calculated at 0.80 and 0.72, respectively (1).

Despite this support for the reliability and diagnostic validity of the CSHQ, a gap in the research literature remains. The construct validity of this measure has never been assessed though direct comparison against the gold standard in sleep measurement (i.e., PSG). One recent study compared CSHQ scores to actigraphy information for 91 typically developing children in an attempt to measure the correlation of this subjective questionnaire to a more objective measure of sleep (24). This study confirmed the presence of several expected associations between the two measures; however, there were also a number of expected hypotheses that were not supported. While this study is the only example, to date, of a comparison between the CSHQ and a more objective sleep measure, there have been mixed findings on the clinical utility of actigraphy in its assessment of sleep. While studies by Lichstein et al. (13) and Vallières and Morin (16) showed support for actigraphy as a satisfactory measure of sleep, results from Sivertsen et al. (15) indicate suboptimal clinical utility. Meltzer et al. (14) found actigraphy was effective at detecting sleep, but significantly overestimated nocturnal awakenings. Furthermore, these same researchers found poor agreement between two different actigraph brands, suggesting that this measure is not universally reliable. Thus, a comparison of the CSHQ to both PSG and actigraphy is needed. The widespread use of the CSHQ as an assessment tool further underscores the importance of evaluating its validity as a measure of children’s sleep.

Building on past validation studies, the current study aimed to assess the validity of the CSHQ as an assessment tool for childhood sleep disorders/problems by directly comparing CSHQ scores to sleep parameters as measured by PSG and actigraphy. As several subscales cannot be assessed directly by PSG or actigraphy (e.g., those that measure anxiety or resistance associated with a child’s bedtime), only four of the eight subscales were considered for analysis: Sleep Onset Delay (i.e., the amount of time it takes a child to fall asleep), Sleep Duration (i.e., the length of time for which the child sleeps), Night Wakings (i.e., any awakenings the child experiences after falling asleep), and Sleep Disordered Breathing (i.e., whether the child has nocturnal breathing difficulties such as snoring or obstructive sleep apnea). Finally, these four CSHQ subscales were assessed for sensitivity and specificity of accuracy in identifying clinically abnormal sleep behavior. The construct validity and diagnostic validity of these four CSHQ subscales were explored as follows.

### Correlation between CSHQ subscale scores and PSG/actigraphy parameters

Scores from the CSHQ Sleep Onset Delay, Sleep Duration, Night Wakings, and Sleep Disordered Breathing subscales were compared to applicable sleep parameters as measured by PSG and actigraphy. Positive correlations were expected for all subscales, with the exception of Sleep Duration. As higher CSHQ scores indicate the presence of problem behaviors, a high Sleep Duration subscale score indicates that a child sleeps too little. As such, scores for this subscale were expected to be negatively correlated with actual sleep duration as measured by both PSG and actigraphy.

### Sensitivity and specificity of CSHQ diagnostic accuracy

To assess the sensitivity and specificity (and thereby the diagnostic validity) of the four previously mentioned CSHQ subscales, clinically significant subscale scores were assessed for each participant. The participants reaching score cutoffs for each CSHQ subscale were compared to those participants reaching PSG and actigraphy cutoffs for related sleep behaviors. This allowed for assessment of whether the CSHQ Sleep Onset Delay, Sleep Duration, Night Wakings, and Sleep Disordered Breathing subscales were able to accurately identify problematic sleep behavior. Based on the overall sensitivity and specificity of the CSHQ as reported by Owens et al. (1), CSHQ subscale scores were expected to accurately identify 80% of children who reached PSG and actigraphy criteria for sleep-disturbed behavior in the current study.

## Materials and Methods

### Participants

Participant data were collected from two larger studies carried out under the direction of Dr. Penny Corkum at Dalhousie University^1^ . These two studies involved identical participant inclusion/exclusion criteria and baseline protocols, and as such, the baseline data from both studies were combined for the current study. Participants included 30 typically developing school-aged children, of which 80.0% were boys (*n* = 24) and 20.0% were girls (*n* = 6). Participants ranged in age from 6.2 to 12.1 years, with a mean age of 8.6 years (SD = 1.7). The sample was primarily Caucasian (93.3%, *n* = 28), with 6.7% (*n* = 2) identified as multi-racial/other. Household income data for all participants were as follows: $21,000–$30,000 (3.3%, *n* = 1), $31,000–$40,000 (6.7%, *n* = 2), $41,000–$50,000 (16.7%, *n* = 5), $51,000–$60,000 (3.3%, *n* = 1), $61,000–$70,000 (16.7%, *n* = 5), and >$71,000 (50.0%, *n* = 15); missing data (3.3%, *n* = 1). Each participant’s socioeconomic status (SES) was determined by selecting the highest of their parents’ occupational scores, calculated as per the Nam-Powers-Boyd scale (25) [*M*_score_ = 71.2, score range: 39–96; missing data (*n* = 1)].

Community recruitment advertisements encouraged interested parents to contact the study’s project coordinator in order to have their children participate in a sleep study. Eligibility was assessed by administering multiple screening questionnaires. Participants met eligibility requirements if they were between 6 and 12 years of age, had no cognitive impairment, and had no prior diagnosis of mental health problems. In addition, eligible participants had no suspected major sleep problems, had no chronic medical/neurological conditions, were never treated with psychotropic medication for a mental health disorder, and had not traveled across more than two time zones in the month prior to participation in the study.

### Procedure

The current research was approved by the IWK Health Centre Research Ethics Board, and was performed in accordance with the Tri-Council Policy Statement for Ethical Conduct of Research Involving Humans. After parental consent, participants were provided with actigraphs and daily sleep diaries. Each participant wore an actigraph, which collected data for a typical week of home sleep/wake behavior. During the same period of time, parents completed the sleep diaries. Following the actigraphy week, participants visited a community hospital for an overnight stay in a child-friendly sleep lab, where continuous PSG recordings were collected for an entire night’s sleep. At this point, parents completed the CSHQ and were instructed to base their ratings on their child’s sleep behavior during the previous week (which corresponded to the time period for which actigraphy recordings were collected).

### Measures

#### Polysomnography

Sandman^®^ Elite SD 32+ Digital Sleep Recording System (Embla Systems, Inc.) was used for recording overnight PSG data using a standard laboratory PSG protocol. PSG recordings were scored by a registered PSG technologist, who was supervised by a physician specializing in sleep medicine. Data included information collected via central and occipital electroencephalography (EEG; i.e., brain activation), left and right electrooculography (EOG; i.e., eye movements), electromyography (EMG; i.e., skeletal muscle activation), and electrocardiography (ECG; i.e., heart rate). In addition, piezo chest bands, nasal cannulas, and finger pulse oximeters assessed respiratory effort, airflow, and oxygen saturation. Furthermore, continuous audio and video recordings were collected during the night in order to increase the ability to detect sleep disturbances (e.g., snoring). The PSG variables included Sleep Latency (i.e., the number of minutes from lights out to sleep onset), Total Sleep Time (i.e., the number of minutes spent asleep in bed, determined as the sum of sleep stages 1 through 4 plus REM sleep), Wake After Sleep Onset (WASO; i.e., the percentage of time spent awake between sleep onset and offset), and a Respiratory Disturbance Index (RDI; i.e., a measure of disordered breathing during sleep, such as obstructive sleep apnea).

#### Actigraphy

Micromini Motionlogger actigraphs (Ambulatory Monitoring Inc.) were provided to participants for estimation of sleep–wake cycles in home settings. For each participant, the actigraph was worn on the wrist of his or her non-dominant hand. The actigraphs were initialized and the data were downloaded using ACTMe Millennium software version 3.47.0.3 (Ambulatory Monitoring Inc.). Actigraph data were collected using zero-crossing mode in 1-min epochs and were scored using Action-W software version 2.6 (Ambulatory Monitoring Inc.). Once downloaded, all actigraph data were scored by the same research assistant. The actigraphy variables collected for the current study included Sleep Latency (i.e., the number of minutes between lights out and sleep onset, with lights out being indicated by parent-completed sleep diaries), Sleep Minutes (i.e., the number of minutes spent asleep while in bed), and WASO (i.e., the percentage of minutes spent awake between the onset and offset of sleep).

#### Children’s Sleep Habits Questionnaire (CSHQ)

The CSHQ is a parent-rated questionnaire comprised of 45 items; 33 scored questions, and 7 additional items intended to provide other relevant information pertaining to sleep behavior (e.g., nocturnal body pains) (1). Each scored question is rated on a 3-point scale as occurring “usually” (i.e., 5–7 times within the past week), “sometimes” (i.e., 2–4 times within the past week), or “rarely” (i.e., never or 1 time within the past week). A number of items on the questionnaire are reverse-scored, so that higher scores consistently indicate problem behaviors. Ratings are combined to form eight subscales: Bedtime Resistance, Sleep Onset Delay, Sleep Duration, Sleep Anxiety, Night Wakings, Parasomnias, Sleep Disordered Breathing, and Daytime Sleepiness.

A Total Sleep Disturbances score is calculated as the sum of all CSHQ scored questions, and can range from 33 to 99. (It is worth noting that two scored items are counted twice in the calculation of two different subscales). A Total Sleep Disturbances score of over 41 indicates a pediatric sleep disorder, as this cutoff has been shown to accurately identify 80% of children with a clinically diagnosed sleep disorder (1).

### Data analyses

A power analysis calculation was performed (α = 0.05, β = 0.20) and determined that observation of moderate effects (i.e., *r* = 0.50) required a sample size of 23 participants, which the current study exceeded. The sample size of 30 was of a sufficient size to observe slightly weaker effects (i.e., *r* = 0.44). Analyses were conducted in IBM SPSS Statistics (version 21.0.0.0, 2012) as follows.

#### Correlation between CSHQ subscale scores and PSG/actigraphy parameters

To determine how CSHQ subscales related to PSG and actigraphy, subscale scores were compared to applicable PSG and actigraphy sleep parameters using Pearson’s correlation analyses. The only CSHQ subscale that was not compared to actigraphy data was Sleep Disordered Breathing, as actigraphy is not able to effectively assess nighttime breathing disturbances.

#### Sensitivity and specificity of CSHQ diagnostic accuracy

Analyses were performed to determine the sensitivity and specificity of the CSHQ Sleep Onset Delay, Sleep Duration, Night Wakings, and Sleep Disordered Breathing subscales. Normative data developed by Owens et al. (1) were used to determine CSHQ subscale cutoff scores for the current sample. A clinically significant score was considered to be one that was >1 SD above the mean score of the Owens et al. norms (i.e., Sleep Onset Delay scores above 1.78, Sleep Duration scores above 4.34, Night Wakings scores above 4.40, and Sleep Disordered Breathing scores above 3.87). For each subscale, participants were separated into CSHQ sleep-disturbed and CSHQ non-sleep-disturbed groups based on these cutoffs.

As well, participants were separated into PSG and actigraphy sleep-disturbed groups, and PSG and actigraphy non-sleep-disturbed groups, using normative data presented by Scholle et al. (26) and Verhulst et al. (27). Clinically significant PSG/actigraphy cutoffs were derived from age-appropriate sample means ±1 SD reported by Scholle et al. as follows: Sleep Latency (45.30 min), Total Sleep Time/Sleep Minutes (450.50 min), and WASO (10.30%). A clinically significant RDI cutoff of 3.37 was derived from the Verhulst et al. age-appropriate sample mean +1 SD. For all variables, clinically significant cutoffs were calculated as 1 SD above the mean, with the exception of Total Sleep Time/Sleep Minutes, for which smaller (rather than larger) values are considered more problematic. In this case, the clinically significant cutoff was calculated as 1 SD below the mean.

If a participant was classified as both CSHQ sleep-disturbed and PSG/actigraphy sleep-disturbed on comparable sleep parameters, this was considered as a true positive. If a participant was classified as CSHQ sleep-disturbed and PSG/actigraphy non-sleep-disturbed, this was considered as a false positive. A false negative constituted a classification of CSHQ non-sleep-disturbed and PSG/actigraphy sleep-disturbed. Finally, a true negative was considered as a concurrent classification of CSHQ non-sleep-disturbed and PSG/actigraphy non-sleep-disturbed.

Comparisons were made between the number of true positives, false positives, false negatives, and true negatives to determine the sensitivity and specificity of each CSHQ subscale. For each subscale, CSHQ sensitivity was calculated as the number of true positives divided by the total number of PSG/actigraphy sleep-disturbed participants. Similarly, specificity was calculated for each subscale as the number of true negatives divided by the total number of PSG/actigraphy non-sleep-disturbed participants. A high level of sensitivity would be indicated if the CSHQ subscale identified a large proportion of PSG/actigraphy sleep-disturbed participants as sleep-disturbed. A high level of specificity would be indicated if the CSHQ subscale identified a large proportion of PSG/actigraphy non-sleep-disturbed participants as non-sleep-disturbed.

## Results

Descriptive statistics for all CSHQ, PSG, and actigraphy variables are included in Tables 1 and 2. Of interest, CSHQ Total Sleep Disturbances scores ranged from 34 to 47 (*M* = 39.00, SD = 3.59), with 33.3% of the sample (*n* = 10) meeting the diagnostic cutoff score of 41 for a sleep disorder, and 66.7% (*n* = 20) falling below this cutoff score. As illustrated in Table 1, the mean CSHQ subscale scores for the current study were almost identical to those reported for the Owens et al. (1) community sample. These normative data were used in the current study to separate participants into CSHQ sleep-disturbed and non-sleep-disturbed groups for each subscale. Participant subscale scores that were >1 SD above the Owens et al. means were considered to be clinically significant, and were thus used to designate participants to the CSHQ sleep-disturbed groups.

**Table 1:** **Descriptive statistics for all variables**.

Measure	Range	*M*	SD	Skewness	Kurtosis
**CSHQ**
Sleep Onset Delay	1–2	1.23 (1.25)^a^	0.43 (0.53)^a^	1.33	−0.26
Sleep Duration	3–6	3.40 (3.41)^a^	0.77 (0.93)^a^	2.06	3.90
Night Wakings	3–7	3.50 (3.51)^a^	1.08 (0.89)^a^	2.59	6.42
Sleep Disordered Breathing	3–5	3.23 (3.24)^a^	0.57 (0.63)^a^	2.43	5.04
**Polysomnography**
Sleep Latency (min)	3.80–74.40	25.42 (21.8)^b^	19.76 (23.5)^b^	1.31	0.99
Total Sleep Time (min)	384.00–599.30	489.55 (512.2)^b^	55.30 (61.7)^b^	0.04	−0.52
Wake After Sleep Onset (%)	1.30–32.10	13.40 (5.1)^b^	7.78 (5.2)^b^	0.36	−0.51
RDI	0.00–0.00	0.00 (1.98)^c^	0.00 (1.39)^c^	–	–
**Actigraphy**
Sleep Latency (min)	2.25–81.00	21.64 (21.8)^b^	17.23 (23.5)^b^	1.85	4.09
Sleep Minutes (min)	288.40–598.50	503.24 (512.2)^b^	65.92 (61.7)^b^	−1.40	2.65
Wake After Sleep Onset (%)	0.30–50.00	11.94 (5.1)^b^	11.22 (5.2)^b^	1.59	3.25

*CSHQ, Children’s Sleep Habits Questionnaire; RDI, Respiratory Disturbance Index*.

*All values represent raw scores. Possible score ranges for each CSHQ subscale are as follows: Sleep Onset Delay (0–3), Sleep Duration (3–9), Night Wakings (3–9), and Sleep Disordered Breathing (3–9)*.

Community sample data from

*^a^Owens et al. (1)*,

^b^Scholle et al. (26), and

*^c^Verhulst et al. (27) included for comparison*.

**Table 2:** **Frequency of participants in sleep-disturbed and non-sleep-disturbed groups**.

Measure	*n*	%
**CSHQ**
Sleep Onset Delay
Sleep-disturbed	7	23.3
Non-sleep-disturbed	23	76.7
Sleep Duration
Sleep-disturbed	3	10.0
Non-sleep-disturbed	27	90.0
Night Wakings
Sleep-disturbed	3	10.0
Non-sleep-disturbed	27	90.0
Sleep Disordered Breathing
Sleep-disturbed	5	16.7
Non-sleep-disturbed	25	83.3
**PSG**
Sleep Latency
Sleep-disturbed	4	13.3
Non-sleep-disturbed	26	86.7
Total Sleep Time
Sleep-disturbed	9	30.0
Non-sleep-disturbed	21	70.0
Wake After Sleep Onset
Sleep-disturbed	18	60.0
Non-sleep-disturbed	12	40.0
RDI
Sleep-disturbed	0	0.0
Non-sleep-disturbed	30	100.0
**Actigraphy**
Sleep Latency
Sleep-disturbed	2	6.7
Non-sleep-disturbed	28	93.3
Sleep Minutes
Sleep-disturbed	6	20.0
Non-sleep-disturbed	24	80.0
Wake After Sleep Onset
Sleep-disturbed	14	46.7
Non-sleep-disturbed	16	53.3

*CSHQ, Children’s Sleep Habits Questionnaire; PSG, polysomnography; RDI, Respiratory Disturbance Index*.

Clinically significant PSG and actigraphy cutoffs were derived from age-appropriate community sample means and SD as reported by Scholle et al. (26) and Verhulst et al. (27). These community sample data are included in Table 1. Compared to the sample means reported by Scholle et al., in general the mean PSG and actigraphy parameters for the current sample indicated slightly more problematic sleep (i.e., higher Sleep Latency, lower Total Sleep Time/Sleep Minutes, and higher WASO). RDI values for the current study were lower than those reported by Verhulst et al., with all participants attaining the lowest possible score for this variable.

### Correlation between CSHQ subscale scores and PSG/actigraphy parameters

Correlations between all CSHQ, PSG, and actigraphy variables are included in Table 3. Although one significant correlation was found between two PSG variables, no significant correlations were observed between any CSHQ and PSG variables. In contrast to the PSG sleep parameters, the actigraphy parameters did share a significant correlation with a CSHQ subscale. Specifically, CSHQ Night Wakings was negatively correlated with actigraphy Sleep Minutes, *r*(28) = −0.42, *p* = 0.02, and was positively correlated with actigraphy WASO, *r*(28) = 0.47, *p* = 0.008. This indicates that parent-reported difficulties with children waking up at night were associated with shorter sleep durations and more night wakings as observed by actigraphy. As with PSG, there was also a significant correlation between two actigraphy variables; however, such correlations among variables on the same measure are not discussed further, as these were not the focus of the current study.

**Table 3:** **Correlations between CSHQ, PSG, and actigraphy variables**.

Variable	CSHQ				PSG				ACT		
	SOD	SD	NW	SDB	SL	TST	WASO	RDI	SL	SM	WASO
CSHQ_SOD	1	0.13	0.26	−0.09	0.31	−0.07	−0.05	^a^	0.02	−0.11	0.20
CSHQ_SD		1	0.13	0.17	0.13	−0.16	0.09	^a^	0.33	0.02	−0.10
CSHQ_NW			1	0.31	−0.19	−0.11	0.17	^a^	−0.17	−0.42*	0.47**
CSHQ_SDB				1	−0.24	−0.09	0.11	^a^	0.06	0.15	−0.04
PSG_SL					1	−0.23	0.11	^a^	0.16	0.25	−0.32
PSG_TST						1	−0.80**	^a^	0.03	0.28	−0.28
PSG_WASO							1	^a^	0.05	−0.21	0.24
PSG_RDI								^a^	^a^	^a^	^a^
ACT_SL								^a^	1	0.06	−0.26
ACT_SM								^a^		1	−0.90**
ACT_WASO								^a^			1

*CSHQ, Children’s Sleep Habits Questionnaire; CSHQ_SOD, CSHQ Sleep Onset Delay; CSHQ_SD, CSHQ Sleep Duration; CSHQ_NW, CSHQ Night Wakings; CSHQ_SDB, CSHQ Sleep Disordered Breathing; PSG, polysomnography; PSG_SL, PSG Sleep Latency; PSG_TST, PSG Total Sleep Time; PSG_WASO, PSG Wake After Sleep Onset; PSG_RDI, PSG Respiratory Disturbance Index; ACT, actigraphy; ACT_SL, ACT Sleep Latency; ACT_SM, ACT Sleep Minutes; ACT_WASO, ACT Wake After Sleep Onset*.

Significant values are shown in boldface. *p < 0.05, **p < 0.01.

*^a^Cannot be computed because PSG RDI variable is constant*.

### Sensitivity and specificity of CSHQ diagnostic accuracy

#### CSHQ Sleep Onset Delay

Table 4 displays sensitivity and specificity data for the four CSHQ subscales as compared to PSG and actigraphy. Regarding Sleep Latency, of the four participants who met PSG Sleep Latency cutoffs for disturbed sleep, CSHQ Sleep Onset Delay scores identified 50.0% (*n* = 2) as sleep-disturbed (i.e., a true positive diagnosis), and 50.0% (*n* = 2) as non-sleep-disturbed (i.e., a false negative diagnosis). Of the 26 participants who met PSG Sleep Latency criteria for non-disturbed sleep, 19.2% (*n* = 5) were identified as CSHQ sleep-disturbed (i.e., a false positive diagnosis), and 80.8% (*n* = 21) were identified as CSHQ non-sleep-disturbed (i.e., a true negative diagnosis). Overall, the CSHQ Sleep Onset Delay subscale displayed sensitivity of 0.50 and specificity of 0.81 in its diagnostic accuracy, as compared to PSG Sleep Latency.

**Table 4:** **Sensitivity and specificity of CSHQ subscales as compared to PSG and actigraphy sleep parameters**.

CSHQ subscale	Polysomnography		Actigraphy	
	Sensitivity	Specificity	Sensitivity	Specificity
Sleep Onset Delay	0.50	0.81	0.50	0.79
Sleep Duration	0.22	0.95	0.00	0.88
Night Wakings	0.11	0.92	0.21	1.00
Sleep Disordered Breathing	Undefined	0.83	^a^	^a^

*CSHQ, Children’s Sleep Habits Questionnaire*.

*^a^Cannot be computed because breathing disturbances are not measured via actigraphy*.

Of the two participants who met actigraphy Sleep Latency cutoffs for disturbed sleep, CSHQ Sleep Onset Delay scores identified 50.0% (*n* = 1) as sleep-disturbed (i.e., a true positive diagnosis), and 50.0% (*n* = 1) as non-sleep-disturbed (i.e., a false negative diagnosis). Of the 28 participants who met actigraphy Sleep Latency criteria for non-disturbed sleep, 21.4% (*n* = 6) were identified as CSHQ sleep-disturbed (i.e., a false positive diagnosis), and 78.6% (*n* = 22) were identified as CSHQ non-sleep-disturbed (i.e., a true negative diagnosis). The results using actigraphy were similar to the PSG findings for Sleep Latency, as overall, the CSHQ Sleep Onset Delay subscale displayed sensitivity of 0.50 and specificity of 0.79 in its diagnostic accuracy, as compared to actigraphy Sleep Latency.

#### CSHQ Sleep Duration

Regarding sensitivity and specificity data for the CSHQ Sleep Duration subscale as compared to PSG, of the nine participants deemed sleep-disturbed according to PSG Total Sleep Time criteria, 22.2% (*n* = 2) were classified as CSHQ sleep-disturbed (i.e., true positive), and 77.8% (*n* = 7) were classified as CSHQ non-sleep-disturbed (i.e., false negative). Of the 21 PSG non-sleep-disturbed participants, 4.8% (*n* = 1) were classified as CSHQ sleep-disturbed (i.e., false positive), and 95.2% (*n* = 20) were classified as CSHQ non-sleep-disturbed (i.e., true negative). When compared to PSG Total Sleep Time, the CSHQ Sleep Duration subscale showed overall sensitivity of 0.22 and specificity of 0.95.

Of the six participants deemed sleep-disturbed according to actigraphy Sleep Minutes criteria, 0.0% (*n* = 0) were classified as CSHQ sleep-disturbed (i.e., true positive), and 100.0% (*n* = 6) were classified as CSHQ non-sleep-disturbed (i.e., false negative). Of the 24 actigraphy non-sleep-disturbed participants, 12.5% (*n* = 3) were classified as CSHQ sleep-disturbed (i.e., false positive), and 87.5% (*n* = 21) were classified as CSHQ non-sleep-disturbed (i.e., true negative). When compared to actigraphy Sleep Minutes, the CSHQ Sleep Duration subscale showed overall sensitivity of 0.00 and specificity of 0.88. The actigraphy results follow the same trend as the PSG results when comparing the sleep duration variables to the CSHQ Sleep Duration subscale.

#### CSHQ Night Wakings

The CSHQ Night Wakings subscale was compared to PSG WASO and actigraphy WASO, respectively. According to the PSG WASO cutoff, 18 participants were classified as sleep-disturbed. Of these, 11.1% (*n* = 2) received a concurrent classification of CSHQ sleep-disturbed (i.e., true positive), whereas 88.9% (*n* = 16) were classified as CSHQ non-sleep-disturbed (i.e., false negative). Twelve participants were considered PSG non-sleep-disturbed, of which 8.3% (*n* = 1) were considered CSHQ sleep-disturbed (i.e., false positive), and 91.7% (*n* = 11) were considered CSHQ non-sleep-disturbed (i.e., true negative). In comparison to PSG WASO, the CSHQ Night Wakings subscale displayed sensitivity of 0.11 and specificity of 0.92.

According to the actigraphy WASO cutoff, 14 participants were classified as sleep-disturbed. Of these, 21.4% (*n* = 3) received a concurrent classification of CSHQ sleep-disturbed (i.e., true positive), whereas 78.6% (*n* = 11) were classified as CSHQ non-sleep-disturbed (i.e., false negative). Sixteen participants were considered actigraphy non-sleep-disturbed, of which 0.0% (*n* = 0) were considered CSHQ sleep-disturbed (i.e., false positive), and 100.0% (*n* = 16) were considered CSHQ non-sleep-disturbed (i.e., true negative). In comparison to actigraphy WASO, the CSHQ Night Wakings subscale displayed sensitivity of 0.21 and specificity of 1.00. This trend again, follows similarly to the PSG WASO findings.

#### CSHQ Sleep Disordered Breathing

Lastly, sensitivity and specificity data for the CSHQ Sleep Disordered Breathing subscale were found as follows: no participants were classified as sleep-disturbed according to the PSG RDI cutoff. Of the 30 participants considered PSG non-sleep-disturbed, 16.7% (*n* = 5) were classified as CSHQ sleep-disturbed (i.e., false positive), and 83.3% (*n* = 25) were classified as CSHQ non-sleep-disturbed (i.e., true negative). Based on these results, the CSHQ Sleep Disordered Breathing subscale had a specificity of 0.83. Since there were no PSG sleep-disturbed participants for this subscale, its sensitivity could not be properly assessed and remains undefined for the current study.

## Discussion

The goal of the present study was to assess the validity of the CSHQ as a screening tool for children’s problematic sleep behavior. Four CSHQ subscales (i.e., Sleep Onset Delay, Sleep Duration, Night Wakings, and Sleep Disordered Breathing) were compared to applicable sleep parameters as measured by PSG and actigraphy in order to explore their construct validity. PSG variables included Sleep Latency, Total Sleep Time, WASO, and RDI, while actigraphy variables included Sleep Latency, Sleep Minutes, and WASO. Pearson’s correlation analyses were performed to assess the construct validity of the CSHQ subscales in relation to similar PSG and actigraphy variables. Surprisingly, no significant correlations were found between CSHQ subscales and applicable PSG sleep parameters, and actigraphy parameters only showed significant correlations with one CSHQ subscale (i.e., Night Wakings). The diagnostic validity of the CSHQ was explored by assessing the sensitivity and specificity of each of these four CSHQ subscales in comparison with identification of problematic sleep behaviors as determined by the gold standard of sleep assessment, PSG. These four CSHQ subscales were also compared to actigraphy, which allowed for an examination of the CSHQ’s ability to identify problematic sleep as determined by an objective measure of sleep taken in the home environment. Overall, when compared against both PSG and actigraphy, the CSHQ subscale scores consistently displayed low sensitivity and high specificity.

These results imply that higher scores on CSHQ Sleep Onset Delay, Sleep Duration, Night Wakings, and Sleep Disordered Breathing subscales do not necessarily align with more problematic sleep parameters as measured by PSG and actigraphy. Instead, the scarcity of significant correlations suggests that scores on these subscales share little to no relation with children’s objectively measured sleep behavior. In this respect, it would appear that the CSHQ may not be the most valid screening tool for children’s sleep problems, as an elevated subscale score may not indicate an actual problem in the area of assessment, and vice versa. Although significant predicted correlations were observed between the CSHQ Night Wakings subscale and applicable actigraphy variables, these results must be interpreted cautiously. Actigraphy is not considered to be a consistently reliable measure of nighttime awakenings, and Sivertsen et al. (15) concluded that actigraphy’s clinical utility is suboptimal due to its difficulty in detecting nighttime wakefulness. In contrast to this under-detection reported by Sivertsen et al., Meltzer et al. (14) found that actigraphy significantly overestimated nighttime wake minutes. This reported inconsistency in actigraphy’s ability to accurately detect nighttime wakefulness might account for the fact that actigraphy WASO (and by association, actigraphy Sleep Minutes) were the only sleep parameters significantly associated with any CSHQ subscale. In this light, one must be cautious in interpreting the significance between these actigraphy scores and CSHQ Night Wakings scores. No other actigraphy variable was significantly correlated with CSHQ subscales, which is in line with the mixed results reported by other studies that have used actigraphy to validate sleep questionnaires [e.g., (12, 23, 24)]. Combined, the current results provide only marginal support for this study’s first hypothesis, as only one CSHQ subscale was significantly associated with any related PSG or actigraphy variables. For the most part, this first hypothesis was unsupported by the current findings.

Based on the accuracy of the CSHQ as reported by Owens et al. (1) during its development, as well as the widespread use of this measure as a screening tool, these results are surprising. Although the current sample size was relatively small, power analyses indicated it was of sufficient size to observe moderate effects and none of the correlations with PSG even approached significance. The fact that no significant relations were found between CSHQ subscale scores and related PSG sleep parameters raises questions of the CSHQ’s clinical usefulness as the sole screening tool for childhood sleep disturbances in the four areas assessed. In order to support the use of any proxy sleep measure, that questionnaire’s scores must be closely related to PSG scores, as this is the gold standard of sleep measurement (4). The lack of significant correlations indicates that the construct validity of these four CSHQ subscales is poor.

In addition to the previously discussed correlations, the diagnostic sensitivity and specificity of these four CSHQ subscales were assessed by comparing participants meeting clinically significant subscale score cutoffs with those meeting clinical cutoffs for related PSG and actigraphy parameters. The actigraphy data supplement the PSG data by providing an estimate of the child’s sleep while in the home environment, as opposed to the sleep lab environment. Based on data reported by Owens et al. (1) during the development of this measure, CSHQ subscales were expected to display sensitivity of 0.80 and specificity of 0.72. The current results indicate that the CSHQ’s sensitivity is quite low in comparison with the diagnostic gold standard. This was found for both PSG and actigraphy, meaning that most of the children who exhibited clinically significant Sleep Latency, Total Sleep Time/Sleep Minutes, and WASO according to PSG/actigraphy were not recognized as such by the CSHQ. This is concerning, when one considers the fact that this questionnaire is used widely to screen for sleep difficulties in children. If one extends the results of the current study to the general population, this means that a large proportion of children with legitimate disordered sleep may show “normal” CSHQ scores and risk going undiagnosed. Without proper identification and diagnosis, problem behaviors cannot be effectively treated. If the CSHQ is being used as the sole diagnostic tool by clinicians, this raises the possibility of a large number of children missing the opportunity for treatment with effective sleep interventions.

In contrast to the low sensitivity displayed by the four CSHQ subscales, the CSHQ displayed a high degree of diagnostic specificity with both PSG and actigraphy, meaning it had a low rate of “misdiagnosis,” or false positives. This may be directly related to the questionnaire’s low sensitivity values; it would seem as though the CSHQ consistently under-reports problem sleep behaviors, whether they exist or not. If a questionnaire’s use consistently results in floor effects, it will neither misdiagnose healthy individuals nor properly diagnose unhealthy individuals. Therefore, although the four CSHQ subscales displayed impressive specificity in the current study, their low sensitivity resulted in low overall diagnostic accuracy for this measure as a whole. As relatively high sensitivity and specificity were expected for all subscales, the current study’s second hypothesis regarding the diagnostic validity of the CSHQ was not supported.

The current study has clear clinical implications, in that caution should be taken when using the CSHQ as the sole screening tool for a child with possible sleep difficulties. The low sensitivity and high specificity of these four CSHQ subscales are of limited usefulness for screening or diagnosing sleep problems. Although the CSHQ will likely not misdiagnose a child with a sleep disorder if one is not present, it will likely miss identifying a sleep problem when one does exist. It is important to note that these results do not negate the validity of parental perception of sleep problems in children. In contrast, they suggest that the CSHQ may not be accurately tapping into parent perception, and as such, problem sleep behaviors are not actually being flagged as problematic by this questionnaire. While this questionnaire may serve as a useful starting point for parents and clinicians to broach the subject of a child’s sleep patterns, it seems that its best use would be to complement other more robust measures of sleep behavior. A multimodal approach may be required in order to gain a comprehensive understanding of a child’s sleep, which could include sleep diaries, questionnaires, actigraphy, parent interview, and PSG where possible and when appropriate to use.

As with any research undertaking, the current study is not without limitations. First, a larger sample would have had increased power to detect more subtle effects. The sex distribution of participants was also uneven due to the nature of the studies that provided the current data. Despite this unequal sex distribution, studies have shown that sex differences in sleep do not emerge until puberty (28) and all of the current participants were pre-pubertal. Exclusion criteria for participants included any previously diagnosed or suspected sleep disorder, and it is possible that by refining the sample in this way, potential variability in the data was lost, which may have altered the pattern of results. Building on the current study, future research could test the validity of the CSHQ in a more heterogeneous population that includes both sleep-disordered and non-sleep-disordered children. It is also worth noting that PSG cutoffs for the current study were based on normative data from Scholle et al. (26), whose sample consisted of children in Germany. It is possible that cultural differences exist between typical German and Canadian patterns of sleep behavior, which would skew the mean sleep behavior data. However, a relative lack of North American normative data that matched the current sample necessitated the use of these norms for reference.

A further limitation in the current study design includes the fact that CSHQ results were based on the week leading up to the child’s visit to the sleep lab, meaning that these scores did not technically encompass the child’s sleep on the night of PSG testing. Although all efforts were made at the sleep lab to replicate typical home sleeping patterns (including bedtimes, wake times, and other pre-bedtime rituals), the sleep lab does constitute a different environment from that in which the child is typically used to sleeping. In comparing typically developing children’s first night in a sleep lab to their usual home sleep, Bessey et al. (29) reported significantly reduced sleep duration and interestingly, significantly reduced WASO in the sleep lab setting. This “first night effect” could have altered children’s sleep for the current study, so that PSG recordings did not accurately reflect the typical sleep at home that was the basis for the parent-rated CSHQ scores. However, the inclusion of the actigraph data mitigates this concern, as these data were collected in the home environment during the same week that the CSHQ respondents were basing their ratings on. Similar comparisons were found between CSHQ and actigraphy that were also found between CSHQ and PSG, lending support for the notion that the sleep lab environment may not have been a major confound within the current research study data.

It is also important to highlight the strengths of the current study. This study compared subjective and objective measures of sleep behavior in school-aged children, and represents the first study to assess one of the most commonly used sleep questionnaires, the CSHQ, against the gold standard of sleep measurement, PSG. Furthermore, although a larger sample size would have had more power to detect subtle effects, the current sample of 30 participants was of a sufficient size to detect moderate effects. A larger sample would not necessarily have made for a more meaningful study, as only moderate or large effect sizes would have actually lent support for the validity of the CSHQ. In addition, although the selective exclusion of children with suspected sleep disorders may mean that the current results apply only to children with typical sleep, there was nevertheless quite a bit of variance in CSHQ, PSG, and actigraphy data. Interestingly, 33.3% of the sample had CSHQ Total Sleep Disturbances scores that exceeded the diagnostic cutoff score of 41, which is in line with current sleep disorder prevalence estimates (10). Finally, and most importantly, the current study brought together parent-rated CSHQ scores and PSG for the first time in order to assess the construct validity and diagnostic validity of four of the CSHQ’s eight subscales. As previously discussed, future research could build on the current study’s design by including a larger and more diverse sample of participants, so that the CSHQ’s validity could be assessed for both typical and disordered sleepers. Regardless of any potential alterations to the design of future studies, it is vital that the current research be replicated.

The current findings underscore the importance of not using the CSHQ as the sole sleep screening tool for children. As previously mentioned, further research is required to determine whether these findings are anomalous. The high prevalence of children not meeting recommended nightly sleep requirements (5, 6, 10) is made even more ominous by evidence for the negative impacts of sleep deprivation on children’s cognitive and emotional well-being (7, 8). Combined, these factors clearly underscore the serious need for a valid tool in the screening and diagnosis of sleep problems in children.

## Conflict of Interest Statement

The authors declare that the research was conducted in the absence of any commercial or financial relationships that could be construed as a potential conflict of interest.

## Footnotes

^1^The first of these was a previously completed study that compared sleep parameters and daytime functioning of typically developing children to those of children with attention-deficit/hyperactivity disorder (ADHD) undergoing a stimulant medication trial. The second is an ongoing study that is comparing sleep parameters and daytime consequences of sleep restriction in typically developing children and children with ADHD.
